# Can Early Omega-3 Fatty Acid Exposure Reduce Risk of Childhood Allergic Disease?

**DOI:** 10.3390/nu9070784

**Published:** 2017-07-21

**Authors:** Elizabeth A. Miles, Philip C. Calder

**Affiliations:** 1 Human Development and Health Academic Unit, Faculty of Medicine, University of Southampton, Southampton SO16 6YD, UK; eam@soton.ac.uk; 2 NIHR Southampton Biomeducal Research Centre, University Hospital Southampton NHS Foundation Trust and University of Southampton, Southampton SO16 6YD, UK

**Keywords:** allergy, asthma, eczema, polyunsaturated fatty acid, omega-6, omega-3, inflammation, eicosanoid, resolution, early life origins

## Abstract

A causal link between increased intake of omega-6 (*n*-6) polyunsaturated fatty acids (PUFAs) and increased incidence of allergic disease has been suggested. This is supported by biologically plausible mechanisms, related to the roles of eicosanoid mediators produced from the *n*-6 PUFA arachidonic acid. Fish and fish oils are sources of long chain omega-3 (*n*-3) PUFAs. These fatty acids act to oppose the actions of *n*-6 PUFAs particularly with regard to eicosanoid synthesis. Thus, *n*-3 PUFAs may protect against allergic sensitisation and allergic manifestations. Epidemiological studies investigating the association between maternal fish intake during pregnancy and allergic outcomes in infants/children of those pregnancies suggest protective associations, but the findings are inconsistent. Fish oil provision to pregnant women is associated with immunologic changes in cord blood. Studies performed to date indicate that provision of fish oil during pregnancy may reduce sensitisation to common food allergens and reduce prevalence and severity of atopic eczema in the first year of life, with a possible persistence until adolescence. A recent study reported that fish oil consumption in pregnancy reduces persistent wheeze and asthma in the offspring at ages 3 to 5 years. Eating oily fish or fish oil supplementation in pregnancy may be a strategy to prevent infant and childhood allergic disease.

## 1. Introduction

Epidemiological studies strongly suggest that early life environmental exposures are important determinants of health and disease in later life [1,2]. Nutrition has been identified as one important exposure that influences early development and later outcomes [3,4]. Considerable development of the human immune system occurs in utero and in the weeks and months after birth [5,6,7], and there is evidence that early immune development can be influenced by nutritional factors [8]. Epidemiological, ecological, and case-control studies have associated differences in the patterns of exposure to omega-6 (*n*-6) and omega-3 (*n*-3) polyunsaturated fatty acids (PUFAs) with differences in the incidence and prevalence of atopic sensitisation or its clinical manifestations (allergies, atopic eczema, hayfever, allergic asthma) [9,10]. A molecular and cellular mechanism has been proposed to explain this association [9,10], thus making a causal relationship between fatty acid exposures and risk of allergic disease. In this article, the mechanisms that are proposed to underlie the causal link between early exposure to *n*-6 or *n*-3 PUFAs and altered risk of developing allergic diseases will be described, as will the literature relating early exposure to the different PUFAs to allergic diseases or to relevant immune outcomes. This is an update of an earlier discussion of this topic [11].

## 2. Polyunsaturated Fatty Acids: Metabolic Relationships, Dietary Sources, and Typical Intakes

There are two main families of PUFAs, the *n*-6 and the *n*-3 families. The simplest members of these families are linoleic acid (18:2*n*-6; LA) and α-linolenic acid (18:3*n*-3; ALA), respectively. LA and ALA cannot be synthesized by mammals, including humans, and so they are described as essential fatty acids. Both are synthesised by plants and are therefore found in plant tissues, like leaves, nuts, seeds, and seed oils. LA is found in significant quantities in many commonly consumed vegetable oils, like corn, sunflower, and soybean oils, and in products made from such oils, like margarines. ALA is found in green plant tissues, in some common vegetable oils, including soybean and rapeseed (canola) oils, in some nuts (e.g., walnuts), and in flaxseeds (also known as linseeds), and flaxseed oil. LA and ALA together contribute over 95% of PUFAs in most Western diets, with LA intake most often being in considerable excess of ALA intake. The intake of LA in Western countries increased greatly over the second half of the 20th century, following the introduction and marketing of cooking oils and margarines as an alternative to animal based fats and spreads [12]. The changed pattern of consumption of LA during the 20th century resulted in a marked increase in the ratio of *n*-6 to *n*-3 PUFAs in the Western diet, with this ratio currently being between 5 and 20 in most Western populations.

**Figure 1:**
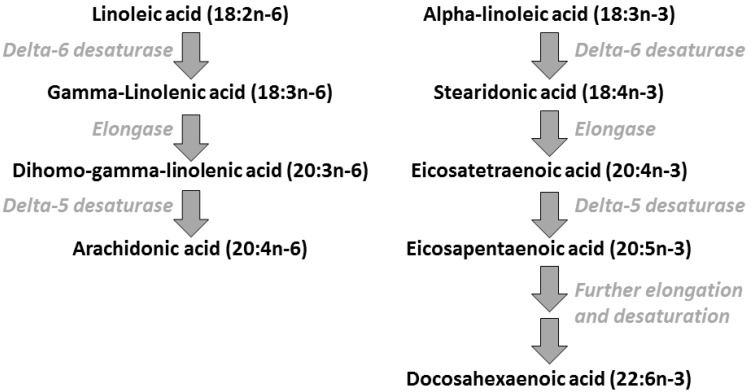
Overview of the pathway of conversion of linoleic and α-linolenic acids to longer chain more unsaturated *n*-6 and *n*-3 polyunsaturated fatty acids (PUFAs).

Although they are not synthesized by humans, LA and ALA can be metabolized to other fatty acids by humans (Figure 1). This metabolic conversion, which mainly occurs in the liver, involves the insertion of new double bonds into the hydrocarbon chain, called desaturation, and the elongation of this chain (Figure 1). This pathway enables the conversion of LA to γ-linolenic acid (18:3*n*-6), di-homo-γ-linolenic acid (20:3*n*-6) and arachidonic acid (20:4*n*-6; AA) (Figure 1). The same pathway and the same enzymes enable the conversion of ALA to eicosapentaenoic acid (20:5*n*-3; EPA). Both AA and EPA can be further metabolised. EPA can be converted to docosapentaenoic acid (22:5*n*-3) and on to docosahexaenoic acid (22:6*n*-3; DHA) (Figure 1). Dietary intakes of AA, EPA, and DHA are much lower than intakes of LA and ALA [13].

In contrast to their precursors, AA, EPA, and DHA are not found in high amounts in plant tissues. Instead, they are found in animal tissues. The most important sources of AA are eggs, meat, and organ meats (offal). The daily intake of AA is estimated to be between 50 and 500 mg among adults in Western countries, with higher intake in people who eat alot of red meat compared to those who do not. EPA and DHA are found in most seafoods, and in the highest amounts in so-called “oily” or “fatty” fish like tuna, salmon, mackerel, herring, and sardines. One serving of oily fish can provide between 1.5 and 3.5 g of EPA plus DHA [13]. A lean fish serving (e.g., of cod) can provide about one tenth of this amount. Fish oil supplements also contain EPA and DHA. A standard fish oil supplement contains about 30% EPA plus DHA; thus, a one gram capsule of such a supplement would contain about 300 mg of EPA plus DHA. In the absence of oily fish consumption or use of fish oil supplements, the dietary intake of EPA and DHA together is likely to be <100 mg/day [13].

## 3. Arachidonic Acid, Lipid Mediators, Inflammation, and Allergic Disease

**Figure 2:**
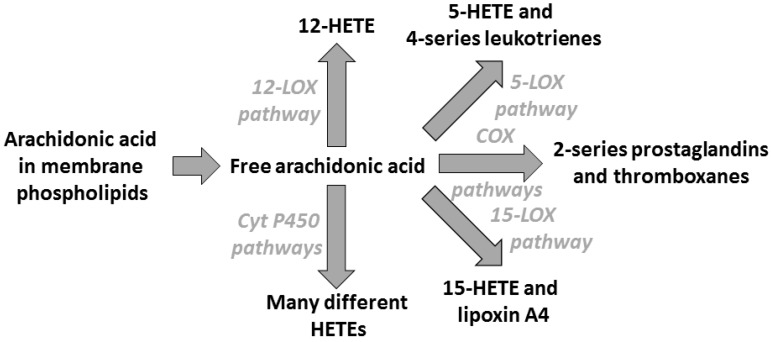
Outline of the pathway of conversion of arachidonic acid to eicosanoids. Abbreviations used: COX, cyclooxygenase; Cyt P450, cytochrome P450; HETE, hydroxyeicosatetraenoic acid; LOX, lipoxygenase.

PUFAs are important components of the phospholipids found in all cell membranes. Phospholipids and their constituent PUFAs play roles in providing the environment that enables membrane proteins to function, by influencing membrane order (“fluidity”) and by promoting specific protein–lipid and protein–protein interactions. As a result of these actions, PUFAs regulate cell signaling, gene expression, and cellular function. Through such membrane-mediated actions, PUFAs can modulate immune cell function [14,15,16], including the inflammatory component [17], and may influence the development and manifestations of allergic diseases [18,19]. However, the key link between PUFAs and the immunological processes related to allergic diseases are the eicosanoids. Eicosanoids are a family of lipid mediators synthesised from 20-carbon PUFAs released from membrane phospholipids upon cell stimulation. Eicosanoids include prostaglandins (PGs), thromboxanes (TXs), and leukotrienes (LTs) (Figure 2). Immune cell membranes usually contain a high proportion of the *n*-6 PUFA AA and low proportions of other 20-carbon PUFAs like EPA. Therefore, the major substrate for synthesis of eicosanoids is usually AA. PGs and TXs are synthesised from the precursor PUFA by the cyclooxygenase (COX) pathway while LTs are synthesised by lipoxygenase (LOX) pathways (Figure 2). The precise mixture of eicosanoids that is produced is determined by the nature, timing, and duration of the initiating stimulus and by the particular cell involved [20,21,22,23]. Some eicosanoids, including PGE_2_, play a role in promoting sensitisation to allergens as a result of their actions on dendritic cells, on T cell differentiation and on immunoglobulin (Ig) class switching in B cells [9,10,18,24]. Other eicosanoids, like the 4-series LTs, are involved in the immunologic features and clinical manifestations of allergic diseases, as a result of their actions on inflammatory, smooth muscle and epithelial cells [9,10,18]. Animal models of allergic inflammation involve increased production of PGs and LTs from AA, suggesting a role of these eicosanoids in the pathology of allergy. However, individual PGs might have different effects, with some enhancing, and others suppressing, allergic inflammation. For example, while PGD_2_, PGF_2α_, and TXA_2_ appear to increase allergic inflammation, PGE_2_ and PGI_2_ appear to inhibit it [25,26,27]. Mast cells and activated macrophages are important sources of PGD_2_. PGD_2_ is a potent bronchoconstrictor, promotes vascular permeability, and activates eosinophils and a pro-allergic Th2-type response [27]. TXA_2_ is a bronchoconstrictor and stimulates acetylcholine release. PGE_2_ is a vasodilator, promotes vascular permeability, inhibits the production of Th1-type cytokines, and primes naïve T cells to produce pro-allergic interleukin (IL)-4 and IL-5 [24]. PGE_2_ also promotes Ig class switching in uncommitted B cells towards the production of pro-allergic IgE [24]. Despite these effects, which are suggestive that PGE_2_ would promote allergic responses, it seems to be protective towards inflammation of the airways [25,26]. It is possible that PGE_2_ has opposing roles, promoting sensitisation via its effects on T cell phenotype and B cells, but protecting against the subsequent manifestations of inflammation upon re-exposure to allergen. PGI_2_ can suppress the activity of Th2 lymphocytes and recruitment of eosinophils, explaining its “anti-allergy” effects. LTB_4_ is chemotactic for leukocytes, increases vascular permeability, induces the release of lysosomal enzymes and reactive oxygen species by neutrophils and of inflammatory cytokines (e.g., tumour necrosis factor-α) by macrophages, and promotes IgE production by B cells. The cysteinyl-LTs (LTC_4_, D_4_ and E_4_) may be either vasoconstrictors or vasodilators depending upon the situation and the location of their synthesis. They cause smooth muscle contraction and bronchoconstriction, and promote vascular permeability, eosinophil recruitment, and mucus secretion. The central role of these eicosanoids in allergic inflammation is indicated by the effective treatment of asthma by LT antagonists. The complex nature of the role of eicosanoids in allergic disease is further illustrated by the interactions that exist amongst these mediators. For example, PGE_2_ inhibits 5-LOX activity so down-regulating LT production [28]. This may be one mechanism by which PGE_2_ is protective towards established allergic disease. Furthermore, PGE_2_ induces 15-LOX, leading to production of lipoxin A_4_ which is anti-inflammatory [29,30,31]. 

**Figure 3:**
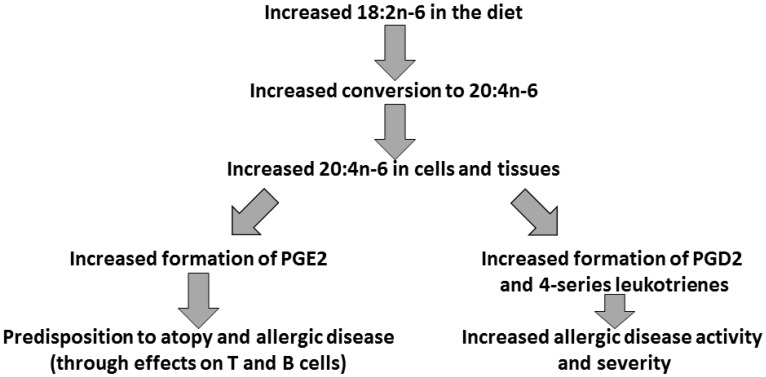
Proposed relationship between increased linoleic acid exposure and increased allergic disease. Abbreviation used: PG, prostaglandin.

The role of AA as the main substrate for the synthesis of eicosanoids and the link between these eicosanoids and inflammation, have lead to suggestions of a causal association between the increased dietary intake of *n*-6 PUFA (mainly as the AA precursor LA) during the second half of the 20th century [12], and the increased incidence and prevalence of allergic diseases over that period [9,10]. The proposed link between dietary *n*-6 PUFA, cell membrane AA, and pro-atopic and pro-allergic eicosanoids is summarised in Figure 3.

In support of the proposed biological mechanism (Figure 3), a high dietary intake of LA has been linked with increased risk of allergic diseases in several studies. Differences in the prevalence of asthma and allergic rhinitis and differences in blood concentrations of allergen-specific IgE in former East and West Germany were related to differences in consumption of LA-poor butter and LA-rich margarine in the two countries [32]. Differences in the prevalence of bronchial asthma, allergic rhinitis, and atopic dermatitis among Finnish schoolchildren were related to levels of LA in plasma cholesteryl esters, an indicator of dietary LA intake [33]. Margarine consumption among German schoolchildren was associated with higher hayfever risk compared with not consuming margarine [34]. Margarine consumption was higher among Australian schoolchildren with atopic dermatitis or with other manifestations of allergic disease compared with controls [35] and high PUFA consumption was associated with increased risk of recent asthma compared with low PUFA consumption [36]. In another study, boys with high margarine consumption were at increased risk of allergic sensitization and of allergic rhinitis compared with those who did not consume margarine [37]. For reasons that are not clear, this relationship was not seen in girls [37]. Swedish children with high consumption of PUFA-rich oils had increased risk of wheeze than those with low consumption [38], while a high dietary *n*-6 to *n*-3 PUFA ratio was associated with increased risk of asthma in Australian schoolchildren [39]. Each of these studies has associated dietary intake and disease at the same point in time. Few studies have attempted to associate early LA exposure to later allergic disease, although there are some studies reporting that LA is higher in breast milk consumed by infants who go on to develop allergic disease in infancy, although not all such studies have found this reviewed in Ref [40]. Furthermore, umbilical cord lipids from neonates who go on to develop allergic disease in early childhood contain a higher amount of LA than normally seen [40], suggesting an early programming effect of higher, compared with lower, LA exposure. A more recent Finnish study reported that a higher ratio of *n*-6 to *n*-3 PUFAs in the diet of pregnant women was associated with higher risk of rhino-conjunctivitis in the offspring at 5 years of age [41].

## 4. Omega-3 Fatty Acids, Lipid Mediators, and Inflammatory Processes

**Figure 4:**
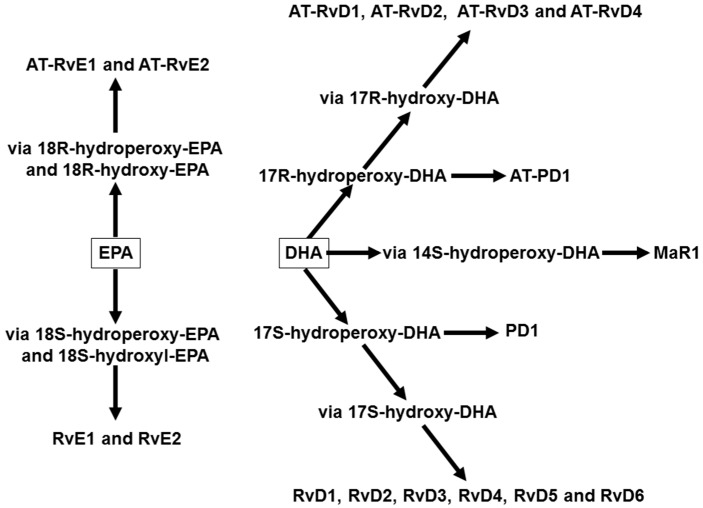
Overview of the pathways of synthesis of specialised pro-resolving mediators from eicosapentaenoic acid (EPA) and docosahexaenoic acid (DHA). Abbreviations used: AT, aspirin-triggered; MaR, maresin; PD, protectin D; Rv, resolvin.

Increased consumption of EPA and DHA (in studies this is usually through use of fish oil supplements) results in enhanced incorporation of EPA and DHA into the phospholipids of immune cell membranes resulting in an elevated proportion of these fatty acids [42,43,44,45]. The incorporation of EPA and DHA into human immune cells is partly at the expense of *n*-6 PUFAs, including AA [42,43,44,46]. This decreases the amount of substrate available for synthesis of 2-series PGs and TXs and 4-series LTs [17,47]. In addition to the reduced production of eicosanoids from AA, EPA is a substrate for COX and LOX enzymes, producing eicosanoids with a slightly different structure to those formed from AA, and the EPA-derived eicosanoids are frequently much less potent than the AA-derived ones [17,47,48]. As an example, LTB_5_ is 10- to 100-fold less potent as a neutrophil chemotactic agent than LTB_4_. In addition to *n*-3 PUFAs decreasing the metabolism of AA to eicosanoids and to EPA acting as substrate for the generation of alternative eicosanoids, another family of lipid mediators is produced from EPA and DHA (Figure 4). This family, termed specialised pro-resolving mediators, includes the D- and E-series resolvins, produced from DHA and EPA, respectively, as well as protectins and maresins produced from DHA. All of these compounds have potent anti-inflammatory and inflammation resolving properties [49,50,51]. 

The role of some resolvins in allergic inflammation has been examined in animal models. Transgenic fat-1 mice can endogenously synthesise *n*-3 PUFAs from *n*-6 PUFAs, a process that is not usually possible in animals [52]. Compared with wild-type mice, fat-1 mice that had been sensitized to ovalbumin had lower infiltration of leukocytes into the airways, lower concentrations of a range of pro-allergic cytokines including IL-5 and IL-13 in lung lavage fluid, increased resolvin E1 and D1 in lung tissue, and showed resistance of the airways to methacholine challenge [53]. These observations suggest that *n*-3 PUFAs might be protective towards allergic inflammation as a result of the synthesis and actions of resolvins. Some other studies have assessed the therapeutic role of resolvins in ovalbumin-sensitised Balb/C mice. In one study resolvin E1 decreased infiltration of eosinophils and lymphocytes into the airways, decreased production of the Th2 cytokine IL-13, lowered circulating ovalbumin-specific IgE concentrations, and reduced airway hyperresponsiveness to inhaled methacholine [54]. In another study, resolvin E1 promoted the resolution of inflammatory airway responses by directly suppressing the production of IL-23 and IL-6 in the lung [55]. More recently resolvin D1 and its epimer aspirin-triggered resolvin D1 were both shown to decrease eosinophil recruitment to the airways, to reduce the production of pro-allergic cytokines and to improve airway hyperresponsiveness to methacholine challenge [56]. These observations suggest that, in contrast to the effects of high intake of *n*-6 PUFAs, a high intake of EPA and DHA will be protective against allergic diseases perhaps acting in part through pro-resolving mediators.

## 5. Omega-3 Fatty Acids and Allergic Disease in Infants and Children

There is some evidence that higher intake of fish, especially fatty fish, in pregnant women is associated with lower risk of allergic disease in the offspring during infancy and childhood [57], but not all studies show this [58]. A Finnish study reported that a low intake of ALA or of total *n*-3 PUFAs in pregnancy was associated with an increased risk of asthma in the offspring at the age of 5 years [59]. Likewise, Miyake et al. [60] found that high maternal ALA intake was associated with reduced risk of wheeze in 16 to 24 months old Japanese children. Pike et al. [61] reported that higher EPA, DHA, and total *n*-3 PUFAs in blood plasma of women in late pregnancy was associated with reduced risk of non-atopic persistent/late wheeze in the offspring. A small number of studies found that infants who go on to develop allergic diseases in infancy consume breast milk with lower EPA and DHA than those who remain healthy, but this finding is not consistent across all studies [40]. Furthermore, umbilical cord blood lipids from neonates who went on to develop allergic disease in early childhood often had lower than normal amounts of EPA and DHA [40]. 

**Figure 5:**
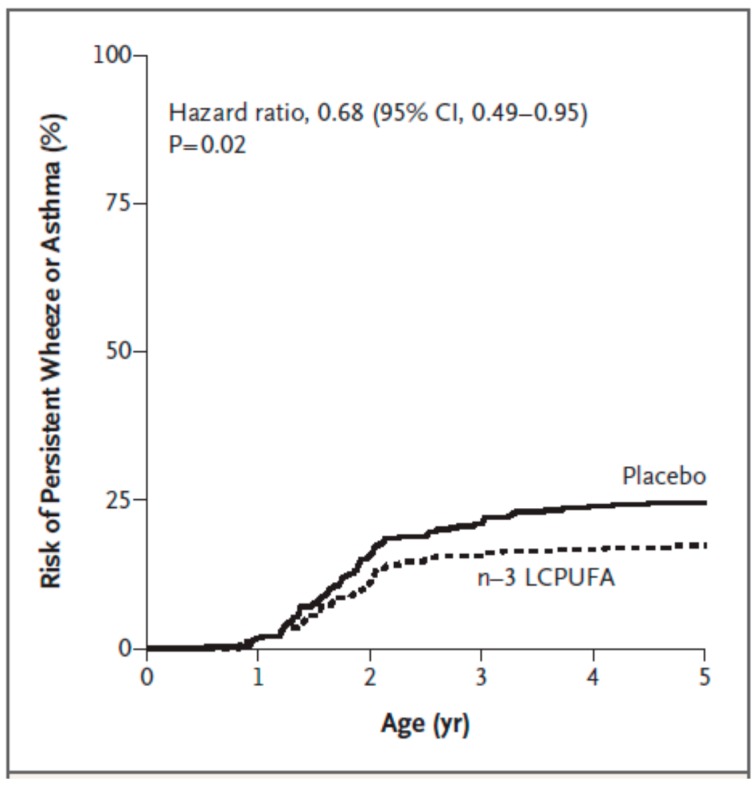
Risk of persistent wheeze or asthma in children according to maternal use of fish oil or placebo during pregnanacy. From New England Journal of Medicine, H. Bisgaard, J. Stokholm, B.L. Chawes, N.H. Vissing, E. Bjarnadóttir, A.M. Schoos, H.M. Wolsk, T.M. Pedersen, R.K. Vinding, S. Thorsteinsdóttir, N.V. Følsgaard, N.R. Fink, J. Thorsen, A.G. Pedersen, J. Waage, M.A. Rasmussen, K.D. Stark, S.F. Olsen, K. Bønnelykke, Fish Oil-Derived Fatty Acids in Pregnancy and Wheeze and Asthma in Offspring, Volume 375, Page 2530–2539. Copyright © 2016 Massachusetts Medical Society. Reprinted with permission from Massachusetts Medical Society.

A small number of studies of maternal fish oil supplementation during pregnancy have been conducted in the context of early immune responses and allergic outcomes in the offspring (studies reporting clinical outcomes [62,63,64,65,66,67,68,69,70] are summarised in Table 1). Several studies have reported that maternal fish oil modifies immune markers in umbilical cord blood [62,71,72,73,74]. These immunologic effects might modify allergic sensitization and the risk of allergic diseases. Indeed, Dunstan et al. [62] reported less severe atopic dermatitis and lower risk of sensitisation to egg in one year old infants whose mothers had consumed fish oil supplements during pregnancy. Some other clinical outcomes were numerically lower in the infants whose mothers had taken fish oil, but the differences were not statistically significant. Olsen et al. [63] reported that fish oil supplementation in late pregnancy was associated with a marked reduction in asthma-related diagnoses in the offspring at age 16 years, suggesting a long term effect of any immunologic changes that occurred in pregnancy and early life. Furthermore, follow-up at age 24 years showed a reduced likelihood of having been prescribed anti-asthma medication in the fish oil group [64], suggesting a long term effect of any immunologic changes that occurred in pregnancy and early life. Fish oil supplementation during both pregnancy and lactation resulted in lower PGE_2_ production by stimulated maternal blood [75], which might influence Th2 polarization in the fetus. In the same study, infants whose mothers had taken fish oil had a lower risk of developing allergic sensitization to egg, less IgE-associated eczema and less food allergy during the first year of life [65]. Over the period of 0 to 24 months there was a lower risk of developing any IgE-mediated disease or IgE-associated eczema or being sensitised to egg or to any allergen that was tested [66]. Palmer et al. [67] found less sensitisation to hens’ egg at age 12 months in offspring of mothers who consumed a DHA-rich oil during pregnancy. There was also a strong trend to less IgE-associated eczema, but there was no difference in “any IgE-mediated disease”. Over the period to age 3 years there was no effect of the DHA-rich oil on any clinical outcome including asthma [68]. However, sensitization to one species of house dust mite was lower at age 6 years [69]. In 2016 Best et al. [76] reported a meta-analysis of offspring clinical outcomes from trials of maternal fish oil supplementation in pregnancy. The results are summarised in Table 2. They identified that maternal fish oil supplementation results in a lower risk of atopic eczema, and less likelihood of having a positive skin prick test to any allergen tested, to hens’ egg, or to any food extract, all in the first 12 months of life. Recently, Bisgaard et al. [70] reported significantly reduced incidence of persistent wheeze or asthma at ages 3 to 5 years in children whose mothers took fish oil during pregnancy (Figure 5). The higher dose of EPA + DHA used, especially of EPA, may explain why these recent findings [70] differ from those of Palmer et al. [67,68] and Best et al. [69]. Furthermore, the studies of Palmer et al. [67,68] and Best et al. [69] reported disease with sensitization (i.e., atopic disease), as opposed the study of Bisgaard et al. [70] which reported wheeze irrespective of sensitization as skin prick testing was only conducted at 6 and 18 months of age. One interesting finding from Bisgaard et al. [70] is that the beneficial effect of maternal EPA + DHA on offspring persistent wheeze or asthma (Figure 5) was seen mainly in the subset of children whose mothers had the lowest EPA + DHA status at study entry, but was less apparent in the subset of children whose mothers had the highest EPA + DHA status at study entry. This observation suggests that *n*-3 PUFAs will be of most benefit to those with the lowest status and may be less effective in those who already have a high status. 

One study has looked at maternal fish oil supplementation during lactation and immune outcomes in the offspring [77]. Mononuclear cells from 2.5 years old children of mothers who received fish oil supplements during lactation produced higher amounts of interferon-γ. This observation was interpreted by the authors to reflect faster maturation of the immune system. Unfortunately, the study did not assess clinical outcomes.

One study has investigated the effect of fish oil given to infants from birth until 6 months of age on immune outcomes [78] and allergic disease [79]. The infants were at high-risk of developing allergy. Mononuclear cells from infants who had received fish oil produced less of the Th2 cytokine IL-13 when stimulated ex vivo with housedust mite [78]. They also produced more of the Th1 cytokines interferon-γ and tumour necrosis factor when stimulated with phytohaemagglutinin. These observations would suggest a favourable shift in the Th1 vs. Th2 balance with fish oil supplementation. The study found that low plasma DHA and low red blood cell EPA were both predictive of eczema by age 12 months [78]. At 12 months of age, clinical outcomes (any allergic disease, total or any specific sensitization, eczema, food allergy, wheeze) were not different between infants who had received fish oil or placebo [79]. However, infants who were most complaint to the intervention had a lower risk of eczema at age 12 months. Furthermore, infants with a higher red blood cell EPA, red blood cell ratio of EPA to ARA or plasma DHA at 6 months of age were less likely to develop eczema by 12 months of age [79]. Infants with higher plasma DHA or EPA + docosapentaenoic acid + DHA at 6 months were less likely to develop recurrent wheeze by 12 months of age [79]. 

One study has examined the long-term effect on allergic diseases of fish oil supplementation of infants [80,81,82,83,84]. There was decreased prevalence of wheeze in the fish oil group at 18 months of age and higher plasma *n*-3 PUFA levels were associated with less bronchodilator use [80,81]. At 3 years of age the fish oil group had reduced cough, but not wheeze and there was no effect of fish oil on other outcomes such as eczema, serum IgE concentration, or doctor diagnosis of asthma [82]. At 5 years of age there was no significant effect of fish oil on any of the clinical outcomes relating to lung function [32], allergy [83], or asthma [84]. Reasons for the lack of beneficial effects of long chain *n*-3 PUFAs at 5 years of age may be suboptimal adherence to the intervention (50% and 56% compliance in the intervention and control group, respectively), the low dose of fish oil used, loss to follow-up, and lack of power. 

Taken together, these studies provide evidence that early exposure to the *n*-3 PUFAs EPA and DHA induces immune effects that may be associated with reduced allergic sensitization and with a reduction in allergic manifestations. However, the data available are not fully consistent and so it is not possible to draw a certain conclusion at this stage. More studies in this area are needed and, where these are interventions, it is important that they be sufficiently powered, that they measure both immune and clinical outcomes where possible, and that dose of *n*-3 PUFAs and duration are carefully considered.

## 6. The Salmon in Pregnancy Study

A systematic review published in 2011 identified that intake of fish, fatty fish, and omega-3 fatty acids in pregnancy is associated with reduced risk of allergic disease in the offspring infants [57]. As with its advice to other adults, the UK Government advises that pregnant women should consume two portions of fish per week, at least one of which should be fatty [85]. The Salmon in Pregnancy Study was a randomised, controlled dietary intervention testing this advice in the context of offspring allergic disease. Pregnant women who were low consumers of fatty fish and who were at risk of giving birth to an infant who would become allergic were recruited [86]. The women were randomized to two groups: one group maintained their habitual diet while the other included salmon twice per week in their diet from week 19 of pregnancy until delivery. Women in the salmon group had a higher dietary intake of EPA and DHA: intake of EPA + DHA from the diet was equivalent to 0.03 g/day in the control group and was 0.4 g/day in the salmon group [86]. Women in the control group showed a decline in the percentage of both EPA and DHA in plasma phosphatidylcholine from week 19 to week 38 pregnancy [86], consistent with other reports [87,88]. However, in the salmon group this decline did not occur and EPA and DHA were seen to increase in plasma phosphatidylcholine over the course of pregnancy [86]. Furthermore, both EPA and DHA were significantly higher in the umbilical cord plasma phosphatidylcholine in the salmon group compared with the control group [86]. Thus, by consuming salmon twice per week mothers were providing more EPA and DHA to their growing fetus. There were also some differences in umbilical cord blood immune cell responses between the two groups, including a lower production of pro-allergic PGE_2_ by cord blood mononuclear cells in response to inflammatory stimuli in the salmon group [89]. Breast milk DHA was higher from women in salmon group at days 1, 5, 14, and 28 after birth, even though women ceased consuming salmon at birth [90]. Thus, women in the salmon group were most likely able to provide a greater amount of DHA to their newborn infant during the early weeks of lactation. Despite, these important findings, at 6 months of age there was no significant difference between the two groups in the number of infants with atopic eczema or in the severity of atopic eczema, in the number of infants showing positive skin prick test responses to common allergens, or in various allergic manifestations [89]. However, the number of infants affected was low in both groups. It is possible that the amount of EPA and DHA provided through two servings of salmon per week (equivalent to 0.4 g/day) was too low to influence the clinical outcomes despite the higher *n*-3 PUFA status in cord blood and the altered cord blood immune cell responses. 

## 7. Summary and Conclusions 

There are two main families of PUFAs, the *n*-6 and the *n*-3 families. Intake of the major plant *n*-6 PUFA LA increased over the second half of the 20th century. This increase in LA intake coincided with increased incidence and prevalence of allergic diseases. A causal link between *n*-6 PUFA intake and allergic disease has been suggested and this is supported by biologically plausible mechanisms, largely related to the roles of eicosanoid mediators produced from the *n*-6 PUFA AA. There is some evidence that high LA intake is associated with increased risk of allergic sensitization and allergic manifestations. Fish and fish oils are sources of the long chain *n*-3 PUFAs EPA and DHA. These fatty acids act to oppose the actions of *n*-6 PUFAs particularly with regard to eicosanoid synthesis. Thus, *n*-3 PUFAs may protect against allergic sensitisation and allergic manifestations. Epidemiological studies investigating the association between maternal fish intake during pregnancy and allergic outcomes in infants/children of those pregnancies suggest protective associations, but findings from these studies are not consistent. Fish oil provision to pregnant women is associated with immunologic changes in cord blood and such changes may persist. Studies performed to date indicate that provision of fish oil during pregnancy may reduce sensitisation to common food allergens and reduce prevalence and severity of atopic dermatitis in the first year of life, with a possible persistence until adolescence. A recent study reported that fish oil consumption in pregnancy reduces persistent wheeze and asthma in the offspring at ages 3 to 5 years. Eating oily fish or fish oil supplementation in pregnancy may be a strategy to prevent infant and childhood allergic disease. Further studies of increased long chain *n*-3 PUFA provision during pregnancy, lactation, and infancy are needed to more clearly identify the immunologic and clinical effects in infants and children and to identify protective effects and their persistence.

## Figures and Tables

**Table 1:** Summary of randomized controlled trials of *n*-3 PUFAs in pregnancy reporting on allergic outcomes in the offspring.

Publication	Particpants	Intervention Details	Outcomes	Differences from Control in *n*-3 PUFA Group
Dunstan et al. [62]	atopic, non-smoking pregnant women (*n* = 98)	fish oil providing 3.7 g *n*-3 PUFAs daily including 1.02 g EPA and 2.07 g DHA; control group received olive oil; from 20 weeks of gestation until delivery	skin prick test positivity (hens’ egg; cows’ milk; peanut; house dust mite; cat), asthma, atopic eczema, food allergy all at 12 months of life	less sensitisation to hens’ egg (odds ratio 0.34; *p* = 0.05); less severe atopic eczema (odds ratio 0.09; *p* = 0.045); less recurrent wheeze, persistant cough and diagnosed asthma but these were not significant
Olsen et al. [63]	pregnant women; *n* = 553	fish oil providing 2.7 g *n*-3 PUFAs daily including 0.86 g EPA and 0.62 g DHA; control group received olive oil; a third group received no intervention; from 30 weeks of gestation until delivery	asthma-related diagnoses at 16 years of life	less incidence of “any asthma” (3.04% vs 8.08%; *p* = 0.03) and “allegic asthma” (0.76% vs. 5.88%; *p* = 0.01)
Hansen et al. [64]	as above	as above	prescription of asthma or allergic rhinitis medication at age 24 years	less prescription of asthma medication (hazard ratio 0.54; *p* = 0.02); trend to less prescription of allerfgic rhinitis medication (hazard ratio 0.70; *p* = 0.10)
Furuhjelm et al. [65]	pregnant women with fetus at high allergic risk; *n* = 145	fish oil providing 2.7 g *n*-3 PUFAs daily including 1.6 g EPA and 1.1 g DHA; control group received soybean oil; from 25 weeks of gestation until 3.5 months post-natally	skin prick test positivity (hens’ egg; cows’ milk; wheat), IgE-antibodies (hens’ egg; cows’ milk; wheat), food allergy, eczema at 3, 6 and 12 months of life	less IgE-associated eczema up to 6 months of life (8% vs. 20%; *p* = 0.06); less IgE-associated eczema (7.7% vs. 20.8%; *p* = 0.02), sensitisation to hens’ egg (11.5% vs. 25.4%; *p* = 0.02), any positive skin prick test (15,4% vs. 31.7%; *p* = 0.04) up to 12 months of life
Furuhjelm et al. [66]	as above	as above	skin prick test positivity (hens’ egg; cows’ milk; wheat; cat; tomothy; birch), food allergy, eczema at 24 months of life	less IgE-mediated disease (11.1% vs. 30.6%; *p* = 0.01), IgE-mediated food reactions (5.6% vs. 21.5%; *p* = 0.01), sensitisation to hens’ egg (13.4% vs. 29.5%; *p* = 0.04), any positive skin prick test (19.2% vs. 36.1%; *p* = 0.048), IgE-associated eczema (9.3% vs. 23.8%; *p* = 0.04) up to 24 months of life
Palmer et al. [67]	pregnant women with fetus at high atopy risk; *n* = 706	fish oil providing 0.9 g *n*-3 PUFAs daily including 0.1 g EPA and 0.8 g DHA; control group received mixed vegetable oils; from 21 weeks of gestation until delivery	skin prick test positivity (hens’ egg; cows’ milk; peanut; wheat; tuna; grass pollen; perennial ryegrass; olive tree pollen; *Alternaria tenuis*; cat; house dust mite), asthma, food allergy, eczema at 12 months of life	less sensitisation to hens’ egg (9% vs. 15%; *p* = 0.02); less IgE-associated eczema (7% vs. 12%; *p* = 0.06)
Palmer et al. [68]	as above	as above	skin prick test positivity (hens’ egg; cows’ milk; peanut; wheat; tuna; cashew; sesame; grass pollen; perennial ryegrass; olive tree pollen; *alternaria tenuis*; cat; house dust mite), asthma, food allergy, allergic rhinitis, eczema at 3 years of life	-
Best et al. [69]	as above	as above	skin prick test positivity (hens’ egg; peanut; cashew; perennial ryegrass pollen; olive tree pollen; *alternaria tenuis*; cat; dog; 2 species of house dust mite), IgE-associated allergic disease symptoms (eczema, wheeze, or rhinitis) with sensitization at 6 years of life	less sensitisation to one species of house dust mite (13.4% vs. 20.3%; *p* = 0.049)
Bisgaard et al. [70]	pregnant women; *n* = 736	fish oil providing 2.4 g *n*-3 PUFAs daily including 1.32 g EPA and 0.89 g DHA; control group received olive oil; from 24 weeks of gestation until delivery	asthma, allergy, eczema; parental report of lung, skin, lower respiratory tract related symptoms; skin prick test positivity (hens’ egg; cows’ milk; cat; dog) at 6 and 18 months of life	less persistant wheeze/asthma from 3 to 5 years of life (hazard ratio 0.68; *p* = 0.02)

**Table 2:** Summary of the findings of the meta-analysis of Best et al. [76] of randomized controlled trials of *n*-3 PUFAs in pregnancy reporting on allergic outcomes in the offspring.

Outcome	Finding (Risk Ratio; 95% Confidence Interval; *p*)	Studies Included
atopic eczema (eczema with positive skin prick test) in the first 12 months of life	0.53; 0.35–0.81; 0.004	[65,67]
any eczema (eczema with or without a positive skin prick test) in the first 12 months of life	0.85; 0.67–1.07; 0.16	[65,67]
cumulative incidence of IgE-mediated rhino-conjunctivitis (rhino-conjuctivitis with a postive skin prick test) in the first 3 years of life	0.81; 0.44–1.47; 0.49	[66,68]
positive skin prick test to any allergen in the first 12 months of life	0.68; 0.52–0.89; 0.006	[62,65,67]
positive skin prick test to hens’ egg in the first 12 months of life	0.54; 0.39–0.75; 0.0003	[62,65,67]
positive skin prick test to any food extract in the first 12 months of life	0.58; 0.45–0.75; <0.0001	[62,65,67]

## Abbreviations

The following abbreviations are used in this manuscript:

AA	Arachidonic acid
ALA	α-Linolenic acid
COX	Cyclooxygenase
DHA	Docosahexaenoic acid
EPA	Eicosapentaenoic acid
Ig	Immunoglobulin
IL	Interleukin
LA	Linoleic acid
LOX	Lipoxygenase
LT	Leukotriene
PG	Prostaglandin
PUFA	Polyunsaturated fatty acid
TX	Thromboxane
